# Vulvar fibroadenoma: a common neoplasm in an uncommon site

**DOI:** 10.1186/1477-7819-7-70

**Published:** 2009-09-28

**Authors:** David Cantú de Leon, Delia Perez Montiel, Hugo Vázquez, César Hernández, Lucely Cetina, Martha Hernandez Lucio

**Affiliations:** 1 Department of Gynecologic Oncology, Instituto Nacional de Cancerologia, Mexico; 2 Department of Pathology, Instituto Nacional de Cancerologia, Mexico; 3 Department of Medical Oncology, Instituto Nacional de Cancerologia, Mexico; 4 Department of Clinical Research, Instituto Nacional de Cancerologia, Mexico

## Abstract

Vulvar fibroadenomas are sporadic lesions informed in the literature and a controversy about origin has been discussed widely. We report a case of a 19 years old woman with a large slow growing mass in the right labia majora with the final diagnosis of fibroadenoma with mammary tissue surrounding it and positive hormone receptors. In this case, we support the origin in ectopic mammary tissue.

## Background

Vulvar lesions in general are infrequent. Malignant neoplasms represent no >5% of gynecological cancers, are more frequent at advanced ages, the most common tumors are epithelial, and among these, epidermoid carcinoma comprises 80% [1]. Mesenchymal neoplasms are even less frequent [2]; vulvar fibroadenoma is one of the mammary-like fibroepithelial lesions of uncertain histogenesis, and is extremely rare [2-7]. These lesions have been reported in the medical literature over the past 50 years [2]. Hartung presented the first description of vulvar mammary tissue in 1872,[8] Bardsley and Petterson made reference to 13 cases in the literature of vulvar mammary tissue-originated primary breast carcinomas,[4] and Yin et al. described the first case of ectopic mammary-tissue mucinous adenocarcinoma in vulva.[9]

At present, controversy exists regarding the histological origin of these lesions. The debate includes the postulation of ectopic mammary tissue-derived lesions, of cutaneous apocrine glands, and mammary-like anogenital glands, the latter the most recent of the theories.[2,3,5,7,10] In the majority of the previous medical literature, ectopic mammary tissue has been postulated as the cause of vulvar and anogenital-region lesions. [2] Aberrant or ectopic mammary tissue occurs in 1-6% of the population and is more frequent upper umbilical scar. [2,6,8,11] Customarily, these are most frequently reported during pregnancy and lactation.[4,6,8] Many previous descriptions of mammary-type lesions in vulva assume their ectopic mammary tissue-derived embryological origin. Nonetheless, documentation of tissue surrounding the lesion has been poor over time with respect to demonstrating healthy mammary tissue in vulva.[3] Ectopic mammary and/or breast-like anogenital gland tissue is subject to hormonal response, because both present hormonal receptors by immunohistochemistry, which leads to the potential of developing benign or malignant processes similar to those observed in normally localized mammary tissue.[3,5-7]

Examples of benign and malignant mammary-type anogenital tumors have been reported sporadically. These tumors are morphologically similar to their mammary counterparts. Among benign lesions are included fibrocystic disease-like changes, intraductal papillomas, fibroadenomas, and phyllodes tumors, while malignant lesions mentioned comprise ductal, lobular, and mucinous adenocarciomas. [2,4,7,9] We present herein the case of a patient with a progressive-growth vulvar lesion with a final report of vulvar fibroadenoma.

## Case presentation

**Figure 1:**
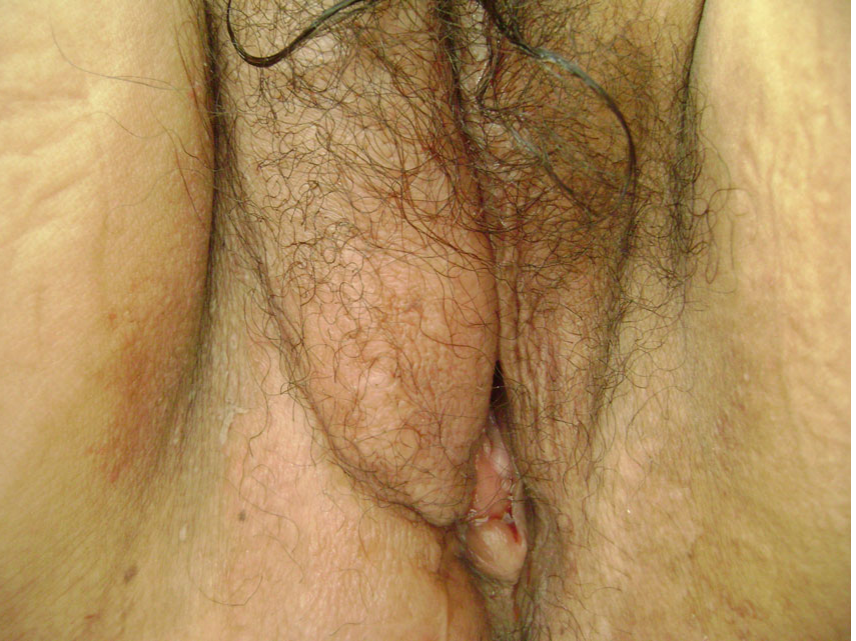
**Tumor in right labium major of the vulva**.

An 18-year-old nulligravida Mexican female was referred to our institution in November 2006 complaining of a vulvar tumor of progressive growth for the previous 12 months. Previous medical and familial history was not contributory to the present illness. Physical examination revealed a 12 × 5-cm tumor located on the right labia majora (Fig 1). The tumor was soft and movable and not adhered to skin or other structures. The remainder of the gynecological, inguinal, and abdominal examination was reported as normal.

Fine-needle aspiration of the lesion was performed, but no cells were obtained. Chest x-ray as well as abdomino-pelvic Computed tomography (CT) scan reported no masses or retroperitoneal lymph node enlargements.

Patient was programmed for wide tumor excision on January 8, 2007. During surgery, the tumor was found as firm, not adhered to adjacent structures, and well circumscribed. Frozen section of the lesion was performed and was reported as benign mesenchymal neoplasm. Primary vulvar-incision closure was performed, and the patient evolved adequately and was discharged 24 h after the surgery. Final pathologic report was ectopic mammary gland-originated fibroadenoma. The patient has been followed up for 17 months and is free of new lesions at present.

## Pathology

**Figure 2:**
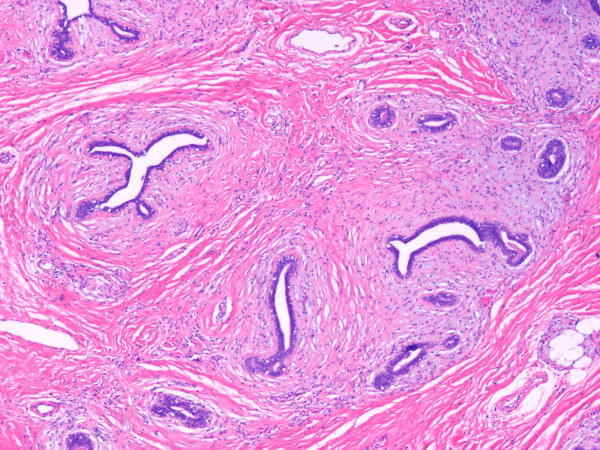
**Microscopic picture showing a low power view of the lesion**. (HE 4 ×).

**Figure 3:**
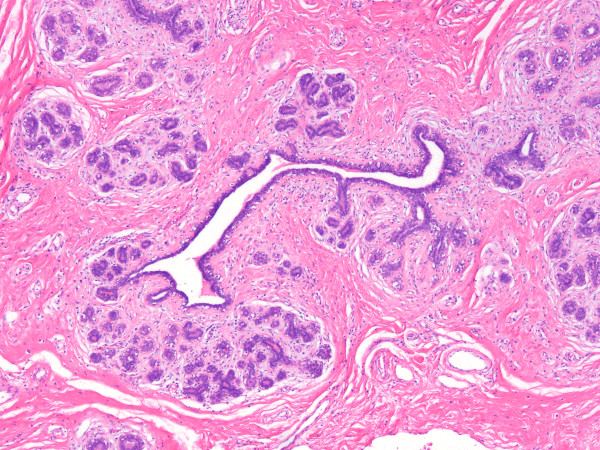
**Microscopic picture of the surrounding tissue of the tumor showing normal breast tissue (HE 20×)**.

Grossly a well delimited multilobular mass with a skin ellipse was received. The measures of the mass was 7 × 4 × 4 cms. Cut surface shows a lobulated white firm mass without necrosis or hemorrhage located in the dermis and subcutaneous tissue no related to skin. Microscopically a fibroepithelial neoplasm with well defined borders was seen; collagenized stroma with more cellular areas around ducts lined by one line of epithelial cells without atypia supported by a layer of myoepithelial cells (Fig 2). Next to this lesion areas of normal breast tissue were present (Fig 3). By immunohistochemical stains the neoplasm was positive to estrogen and progestagen receptors.

## Conclusion

In 2006, Atwal published a case of previously documented supernumerary mammary tissue-originated vulvar fibroadenoma, describing a lesion that histopathologically mimicked a fibroadenoma with positive estrogenic receptors by immunohistochemistry and with healthy mammary tissue surrounding the lesion.[3] The presence of ectopic mammary tissue of normal characteristics surrounding a lesion described as fibroadenoma supports the theory of ectopic mammary tissue, and concludes that not all fibroadenomas derive from anogenital glands similar to breast, as Van der Putte confirmed. [12-14]

Carter in 2008 presented an analysis of 18 reports of prior cases of vulvar fibroepithelial neoplasms, showing an average patient age at moment of diagnosis and surgical extirpation of 38.7 years (range, 20-60 years), average tumor size was 3.0 cm (range, 0.8-6.0 cm). Difference in tumor size and age at diagnosis of phyllodes tumor and fibroadenoma was not significant. Two cases of bilaterality were reported: one of fibroadenoma, and the other, phyllodes tumor. [2] On the other hand, in 2007, Ahmed in his review describes 10 cases of the literature presenting seven as vulvar and three as anogenital lesions (patient age range, 35-84 years). One male was described as among these patients. Tumor size presentation ranged from 0.7 cm-6.0 cm.[7]

Although in the majority of cases ectopic mammary-tissue origin is assumed, only two cases were documented of lesion- or peripheral-associated mammary tissue, these being phyllodes tumors. In no case does the study describe mammary-like anogenital glands. Lack of documentation on vulvar lesion-adjacent tissue can be a limitation for determining reliable lesion histogenesis. The well-circumscribed nature of the lesion permits its simple excision, which implies the need for a more extensive resection for adequate histological review of the surrounding tissue.

We conclude that mammary-type vulvar fibroepithelial-lesion histogenesis remains uncertain. The debate will continue until adequate study is conducted of vulvar lesion-surrounding tissue; its clinical presentation and subsequent behavior are comparable with its counterpart in breast. We should consider in a reserved fashion the publication of Atwal et al. [3] with regard to the theory of Van der Putte [12-14] until the authors describe more cases entailing the same characteristics. The results of the Carter et al. [2] review in the literature in which it is clearly established that vulvar lesion-adjacent tissue was not studied in the majority of cases; thus, it was not established whether ectopic mammary tissue exists, nor was the presence of mammary-like anogenital glands corroborated. We should consider this lesion type within the differential diagnosis of vulvar pathology regardless of the woman's age. Excisional treatment appears to be effective, with low recurrence rates,[2,4,5,8] although the literature includes one case of recurrence, specifically on presenting bilaterally and with phyllodes histology. [2]

Our case increases the number of cases that support the origin in ectopic mammary tissue since we were able to find normal mammarian tissue surrounding the neoplasm and has positive for estrogen and progesterone receptors.

## Consent

Written informed consent was obtained from the patient for publication of this case report and any accompanying images. A copy of the written consent is available for review by the Editor-in-Chief of this journal.

## Competing interests

The authors declare that they have no competing interests.

## Authors' contributions

DCL was responsible for the design and writing of the manuscript. DPM was responsible for the pathologic evaluation and writing of the manuscript. HV was responsible for the literature and case review. CH was responsible for the literature review and writing of the manuscript. LC was responsible for the manuscript completion and critical review. MHL was responsible for the coordination and helped to draft the manuscript. All authors read and approved the final manuscript.
